# Morphological and Molecular Identification of *Lethocerus patruelis* (Stål, 1854) (Hemiptera: Belostomatidae) Specimen Collected in Close Proximity to Humans in Southern Italy

**DOI:** 10.3390/insects16020226

**Published:** 2025-02-19

**Authors:** Donato Antonio Raele, Maria Grazia Cariglia, Stefania Patrizia Grimaldi, Antonella Carla Dinoi, Ettore Franco, Maria Assunta Cafiero

**Affiliations:** 1 Istituto Zooprofilattico Sperimentale della Puglia e della Basilicata, 71121 Foggia, Italy; donatoantonio.raele@izspb.it (D.A.R.); stefania.grimaldi@izspb.it (S.P.G.); 2 Azienda Sanitaria Locale Taranto, Servizi Veterinari, 74024 Taranto, Italy; antonellacarla.dinoi@asl.taranto.it (A.C.D.); ettore.franco@asl.taranto.it (E.F.)

**Keywords:** *Lethocerinae*, aquatic insects, freshwater habitats

## Abstract

The Belostomatidae is a family of aquatic Heteropteran insects that normally inhabit swamps of tropical, subtropical and temperate regions where they naturally feed on a large range of prey, mainly fish, amphibians and reptiles. However, these arthropods can occasionally bite humans when provoked, causing pain. The only species of giant water bug known to be found in Europe is *Lethocerus* (L.) *patruelis*. We reported the finding of a specimen of female *L. patruelis* in Southern Italy. The arthropod was collected by a tourist on his body while he lying down on a beach close to a nature reserve. To finalize the giant water bug identification, both morphological and molecular tools were used for the first time in our country. Herein, information on cases of patients bitten by giant water bugs belonging to the Belostomatidae family are reported, and circumstances involving their contact with humans are also discussed.

## Simple Summary

*Lethocerus patruelis* (Hemipera: Belostomatidae) is the only species of giant water bug known to be present in Europe, including Southern Italy, where it is mainly recorded in areas close to the Adriatic coast. Members of the Belastomatidae family can occasionally bite humans when disturbed. For the first time in Italy, a specimen was collected in a populated area of the Apulia region and identified using morphological and molecular tools. This and previous records provide a reason to draw attention to its presence in Italian tourist places, but actually, it is not really cause for alarm.

## 1. Introduction

Belostomatids are a group of predaceous and aquatic hemipterans that include the largest—up to 120 mm long—representatives of Heteroptera, also known as giant water bugs, electric-light bugs or tadpole killers and water cockroaches [1,2]. These insects are widely distributed in tropical, subtropical and temperate regions [3] and live in limnic environments such as rivers, streams, lakes and ponds. They play a role as bioindicators, predators and biocontrol agents of mosquito’s larvae and snails [4,5]. In different Asiatic countries, some Belostomatidae species are important edible insects [6].

The family Belostomatidae includes about 170 species and it is subdivided into the subfamilies Belostomatinae, with eight genera, and Lethocerinae, with three genera [5]. The latter family includes the *Lethocerus* (Meyr, 1853) genus represented in Europe, including European Turkey and some Greek islands by only one *Lethocerus* (L.) *patruelis* species [7,8,9,10]; more recently, this non-native insect was also recorded in Cyprus [11]. *Lethocerus patruelis*, as well as the other members of this family, naturally catch a large range of prey, such as small reptiles, amphibians, fish, birds and insect larvae, including mosquitoes [12]. The prey is quickly captured and immobilized under water with hooked limbs, and it is subsequently penetrated using a robust chitinous rostrum. Then, the prey is injected with a digestive saliva that contains several enzymes with paralyzing agents [13,14]. Although members of the Belastomatidae family are currently not considered arthropods of medical interest and not mentioned in medical texts and parasitology manuals in relation to human injuries, some species can occasionally inflict bites to humans [15]. Furthermore, water bugs of the Belostomatidae family have been considered to cause insect-mediated infection with pathogens close to aquatic ecosystems, such as *Mycobacterium ulcerans* [16]. Herein, we describe a case of finding a belostomatid specimen on a man, and we provide information on the status and potential role of these insects in human-frequented areas in Italy.

## 2. Materials and Methods

In late August of 2023, the Local Health Agency (ASL) in the Taranto municipality (Italy, the Apulia region) delivered a plastic box to the Istituto Zooprofilattico di Puglia e Basilicata, which contained a big bug stored in alcohol at 70%, suspected to be dangerous to humans, with an identification request. The anamnesis detailed that the arthropod was promptly collected while it was on the foot of a 48-year-old man, who was lying down on a local beach very close to a nature reserve. Very alarmed, the man collected the insect using a beach towel, put it in a plastic container and delivered it to the Veterinary Services of the local ASL. The identification was performed according to Novoselsky’s morphological keys [17] and confirmed by molecular methods. The total genomic DNA was extracted with the commercial GeneJET Genomic DNA Purification Kit (Thermo Scientific, Vilnius, Lithuania) from the specimen’s visceral component. DNA was used as a template in a PCR targeting the mitochondrial cytochrome c oxidase subunit I (*COX1*) according to Folmer’s procedure [18], and PCR products were electrophoresed on 2% agarose gel and stained with Sybr^®^ Safe (Thermo Scientific, Milan, Italy). A standard UV transilluminator (Bio-Rad, Milan, Italy) was used to visualize the separated products. The amplicon was purified by the GeneJET PCR Purification Kit (Thermo Scientific) and was sent to Eurofins Genomics (Milan, Italy) to determinate its nucleotide sequence using the Sanger technique, sequencing PCR products via the Big Dye Terminator Kit (Thermo Scientific). Assays were performed strictly according to the manufacturer’s instructions. The final sequence was assembled and submitted to GenBank with the accession number OR864365.1. BLAST (https://blast.ncbi.nlm.nih.gov/Blast.cgi, accessed on 4 December 2023) was used to compare this sequence to others in GenBank, and the corresponding sequences were chosen and aligned with the one from this study using the ClustalW algorithm [19]. The phylogenetic tree for *Lethocerus* spp. was constructed using maximum likelihood (ML) estimations based on the cytochrome *C oxidase subunit 1* (*COI*) gene (501 model). Branch numbers represent percentage bootstrap support (1000 replicates), with only supports greater than 75% shown. The belostomatid *Appasus japonicus* (Vuillefroy, 1964) species was applied as an outgroup sequence.

## 3. Results

**Figure 1:**
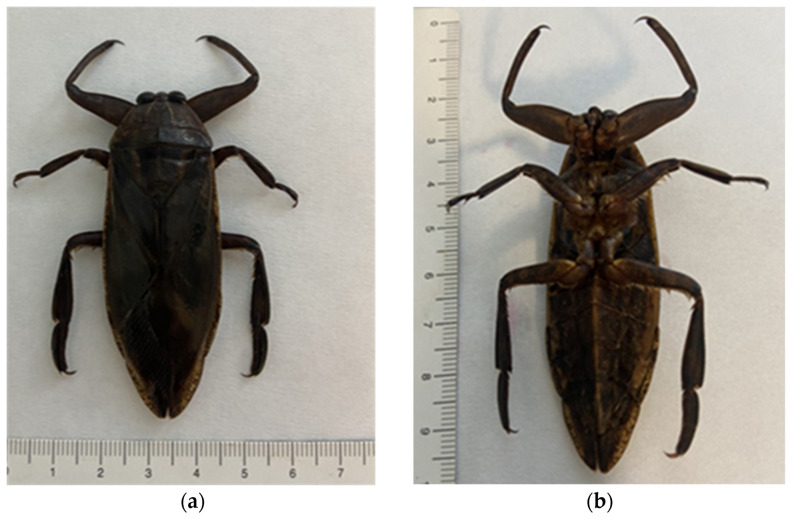
*Lethocerus* (L.) *patruelis*: (**a**) dorsal view, (**b**) ventral view.

**Figure 2:**
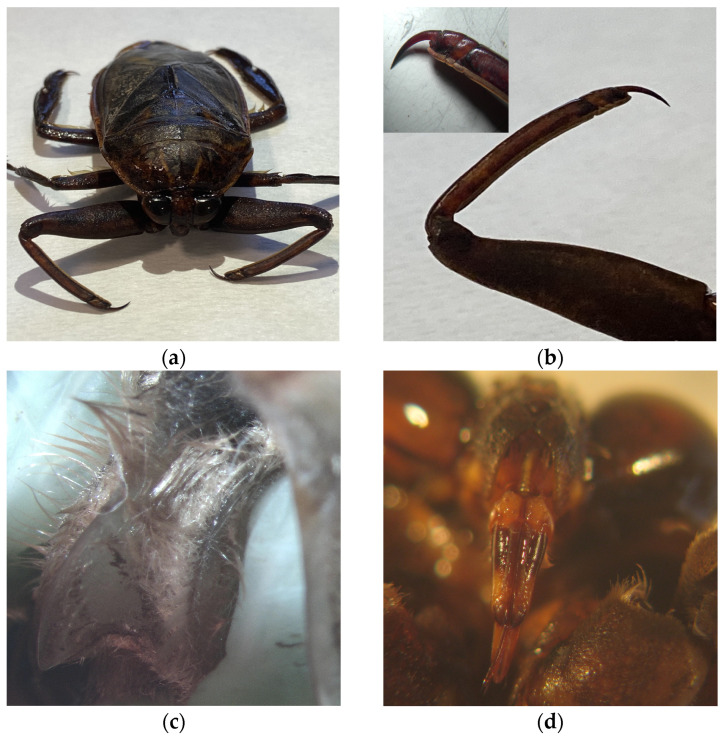
Morphological characteristics useful for the identification of the *L. patruelis* specimen. (**a**) Pronotum with two narrow divergent light stripes, (**b**) three-segmented foretarsus, (**c**) prosternum medially (**d**) rostrum.

**Figure 3:**
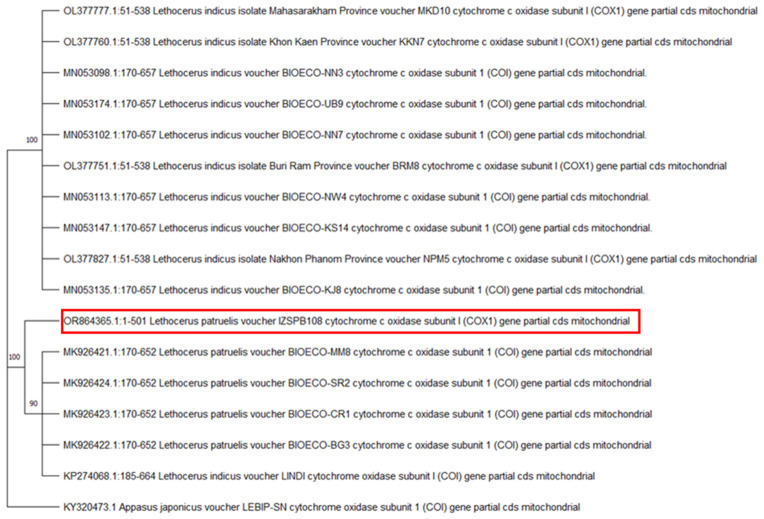
The phylogenetic tree for *Lethocerus* spp. is constructed using maximum likelihood (ML) estimations based on the cytochrome C oxidase subunit 1 (COI) gene (501 unambiguously aligned bp; best fit approximation for the evolutionary model: the Tamura-Nei model). The phylogenetic tree includes representative Belostomatidae species, with their GenBank accession numbers indicated. Branch numbers represent percentage bootstrap support (1000 replicates), with only supports greater than 75% shown. The scale bar represents units of substitution per site. The red square denotes the *L. patruelis* sequence obtained in this study.

According to Novoselsky’s morphological features [17], the specimen was identified as a female *Lethocerus patruelis* (80 mm long and 20 mm large) (Figure 1 and Figure 2). A fragment of the cytochrome c oxidase subunit I (*COX1*) gene (710 base pairs [bp]) was successfully amplified (accession number OR864365.1). The subsequent molecular analysis and the BLAST analysis showed that the yielded amplicon was 98,96% identical to the corresponding region of the NCBI sequences of the *L. patruelis* mitochondrial partial *COX1* gene. The phylogenetic analysis showed that the obtained sequence clusters with those of *L. patruelis* linked to the Asian group, except for only one sequence, KP274068.1, referred to as *L. indicus* (Figure 3) (Table 1).

## 4. Discussion

*Lethocerus patruelis* is the only European Belostomatidae species and the largest verified bug in this continent. This non-native insect is widely distributed in south-eastern European countries, mainly within the Balkan Peninsula. In recent decades, this arthropod has been recorded more times in Southern Italy in localities close to sea ports and nature reserves [9,20,21,22,23,24]. It is assumed that the distribution of *L. patruelis* in certain Italian areas may be linked to marine traffic [9], likely reflecting a westward expansion [20]. In fact, adults are usually attracted by lights, including those of ships; this behavior may have favored the repeated introduction of individuals from the Balkan countries to localities close to the Adriatic coasts (the Abruzzi and Apulia regions), where favorable ecoclimatic conditions could also permit the reproduction of *L. patruelis* [20].

In Southern Italy, the presence of L. patruelis adults of both sexes in immature stages has not been registered so far; its reproduction in Italy has not been confirmed [24,25]. The increasing records of the insect’s presence in Southern Italy suggest that greater attention should be devoted to this issue due to the ability of members of the Belostomatidae family to bite humans when accidentally provoked. However, because they are not specifically arthropods of medical interest, information on this topic remains poor and limited to very sporadic medical reports. Particularly, Haddad reports seven episodes of human attacks by Belostomatidae from Brazil, occurring in ichthyologists/individuals working/playing in small rivers and ponds inhabited by giant water bugs [15]. In all the cases, a red point was visible on the bite sites, mostly localized on hands and fingers but also on the forearm and foot. The observed clinical manifestations varied from 1 to 5 h, and they range from mild to intense edemas with severe, excruciating pain sometimes referred as pulsatile; transitory anesthesia followed by paresthesia of the forearm was also reported in a zoologist attacked by *Lethocerus delpontei* (De Carlo, 1930). In the remaining described cases, insect identification was restricted to the level of the Belostomatidae family.

In Europe, only one case of a human puncture by a member of the Belostomatidae family was observed in Italy in 2010, precisely in the Apulia region in a locality close to the Adriatic Sea. The attack involved a woman bathing herself in the sea. Intense pain in the bite site and transitory and mild fever (37.5 °C) was registered in the patient for few hours [26]. The case was attributed to member of the genus *Lethocerus,* but it is probable that the involved species was *L. patruelis*, the only confirmed taxon of the European Belostomatidae family and reported more than fifty times in the same Apulia region where its potential suitable habitats are located (wetlands and aquatic environments) [9,26]. Evidence shows that some species (*Lethocerus*) appear to be able to live in seawater—at least for a while [27].

Although proof of its naturalization has yet to be found, data suggest that a viable population of *L. patruelis* could possibly exist in Southern Italy [22]; consequently, the encounter of this alien insect with man may not be a remote possibility in this area. For this reason, in addition to the literature, data could be very useful to acquire more information on the presence and diffusion of this belostomatid arthropod through unconventional data sources, such as citizen science, online forums, etc., as suggested by Lo Parrino and Tomasi [25] and confirmed by recent experiences [11].

Belostomatids naturally prey upon small vertebrate living in freshwater habitats; they are currently not aggressive and they pose no threat to humans [12]. However, because the encounter between this insect and man may accidentally result in a puncture, the sightings of *L. patruelis* should receive adequate attention from health services in addition to entomologists, mainly in certain areas of the Adriatic coast, such as what was shown in the reported case herein. Furthermore, belostomatids are also very large insects, and even just one encounter with them could raise alarm in tourist localities and in the summer season. In fact, in Southern Italy, a high number of reports of *L. patruelis* occur in August through September in proximity of seaside areas [25], such as that observed in the described episode. Summer clothing exposing the body, especially areas such as the feet, can make humans more vulnerable to punctures due to an unexpected attack from this insect. Unsurprisingly, members of the Belastomatidae family have been dubbed “toe-biters” because they can produce excruciating bites if inadvertently experienced by the bare-footed [28].

The *Lethocerus patruelis* specimen examined in this study is included in the Asiatic cluster of *L. patruelis.* To date, only African Belostomatidae exhibit persistent colonization with *Mycobacterium ulcerans*, an environmental pathogen causing *Buruli ulcer* disease, a debilitating cutaneous disease that mainly spreads in West Africa, and they may have a role in the epidemiology of this infection [29]. The first documented case of *Buruli ulcer* disease following a bite from a water bug from the Belostomatidae family seems to strongly demonstrate the ability of such insects to act as vectors of the *M.ulcerans* pathogen [16]. A molecular approach for belostomatids may provide both the correct identification and useful information on the possible origin of the collected specimens, and it is used herein for the first time in Italy. However, the study of more genes is necessary to better understand the possible origin of the collected specimens and their role. The described case and recent findings of *L. patruelis* located close to humans in the Mediterranean area, including Italy, add information on possible human exposure to this arthropod in wetlands favorable to its colonization [11,21,23]. Although this insect is usually not included in the arthropod species of medical interest, its capacity to carry infections (i.e., *Buruli ulcer*) and inflict very painful lesions should lead to a reconsideration of its role in health. Studying areas with the previous presence of the giant water bug may also be useful in providing information to local health services to help us consider it as a remote but possible cause of human dread and injuries when carelessly handled or accidently disturbed.

## Figures and Tables

**Table 1:** Based on the analyzed data, the table shows the GenBank association number, sites of the collected species and BLAST% identity. * *L. indicus*, dubious assignment.

	***L. patruelis***					***L. indicus***											***A. japonicus***
	**OR864365^§^**	**MK926421**	**MK926422**	**MK926423**	**MK926424**	**OL377777**	**MN053098**	**OL377760**	**MN053174**	**MN053102**	**MN053113**	**MN053147**	**MN053135**	**OL377751**	**OL377827**	KP274068 *	**KY320473**
OR864365^§^	100%	98.76%	98.76%	98.96%	98.96%	89.00%	89.41%	89.00%	89.61%	89.61%	89.41%	89.61%	89.82%	89.41%	89.61%	98.96%	80.34%
MK926421		100%	99.70%	99.85%	99.85%	89.22%	90.26%	89.22%	90.26%	90.26%	90.26%	90.34%	90.56%	89.59%	89.78%	99.85%	80.54%
MK926422			100%	99.85%	99.85%	88.85%	89.94%	88.85%	89.95%	89.95%	89.95%	90.03%	90.26%	89.22%	89.41%	99.85%	80.54%
MK926423				100%	100%	89.03%	90.11%	89.03%	90.11%	90.11%	90.11%	90.18%	90.41%	89.41%	89.59%	100%	80.70%
MK926424					100%	89.03%	90.11%	89.03%	90.11%	90.11%	90.11%	90.18%	90.41%	89.41%	89.59%	100%	80.70%
OL377777						100%	99.26%	99.63%	99.07%	98.89%	99.26%	99.07%	98.89%	99.26%	99.07%	89.06%	80.70%
MN053098							100%	99.26%	99.39%	99.39%	99.39%	99.54%	99.39%	99.63%	99.44%	90.14%	80.38%
OL377760								100%	99.07%	98.89%	99.26%	99.07%	98.89%	99.26%	99.07%	89.06%	80.70%
MN053174									100%	99.70%	99.39%	99.54%	99.39%	99.81%	98.63%	90.14%	80.38%
MN053102										100%	99.39%	99.54%	99.39%	99.63%	99.44%	90.14%	80.38%
MN053113											100%	99.54%	99.39%	100%	98.81%	90.14%	80.70%
MN053147												100%	99.54%	99.81%	99.63%	90.14%	80.54%
MN053135													100%	99.63%	99.81%	90.45%	80.70%
OL377751														100%	99.81%	89.43%	80.90%
OL377827															100%	89.62%	80.90%
KP274068 *																100%	80.56%
KY320473																	100%
**COI GenBank Association No.**			**Species**					**Collecting Site**					**BLAST % Identity**				
MK926421			*L. patruelis*					Myanmar					98.76%				
MK926422			*L. patruelis*					Bulgaria					98.76%				
MK926423			*L. patruelis*					Thailand					98.96%				
MK926424			*L. patruelis*					Thailand					98.96%				
OL377777			*L. indicus*					Thailand					89.00%				
MN053098			*L. indicus*					Thailand					89.41%				
OL377760			*L. indicus*					Thailand					89.00%				
MN053174			*L. indicus*					Thailand					89.61%				
MN053102			*L. indicus*					Thailand					89.61%				
MN053113			*L. indicus*					Thailand					89.41%				
MN053147			*L. indicus*					Thailand					89.61%				
MN053135			*L. indicus*					Thailand					89.82%				
OL377751			*L. indicus*					Thailand					89.41%				
OL377827			*L. indicus*					Thailand					89.61%				
OR864365^§^			*L. patruelis*					Italy					100%				
KP274068 *			*L. indicus*					India					98.96%				
KY320473			*Appasus japonicus*					Japan					80.34%				

## Data Availability

Data are available at https://www.ncbi.nlm.nih.gov/nuccore/OR864365.1, accessed on 3 December 2023.
